# A preliminary randomized trial of the safety, tolerability, and clinical effects of hemp-derived cannabidiol in alcohol use disorder

**DOI:** 10.3389/fpsyt.2025.1516351

**Published:** 2025-04-28

**Authors:** Raeghan L. Mueller, Jake F. Hooper, Jarrod M. Ellingson, Aviva K. Olsavsky, Rachael Rzasa-Lynn, Angela D. Bryan, L. Cinnamon Bidwell, Kent E. Hutchison

**Affiliations:** ^1^ Department of Psychiatry, University of Colorado Anschutz Medical Campus, Aurora, CO, United States; ^2^ Children’s Hospital Colorado, Aurora, CO, United States; ^3^ Department of Anesthesiology, University of Colorado Anschutz Medical Campus, Aurora, CO, United States; ^4^ Department of Psychology and Neuroscience, University of Colorado Boulder, Boulder, CO, United States

**Keywords:** cannabidiol, thc, cannabinoids, alcohol use disorder, alcoholism, clinical trial

## Abstract

**Introduction:**

Cannabidiol (CBD) has recently gained attention for its potential therapeutic effects in substance use disorders, including Alcohol Use Disorder (AUD). This study examined the potential therapeutic effects of commercially available products containing CBD with and without a small amount of tetrahydrocannabinol (THC) on alcohol use and craving among individuals with moderate to severe AUD.

**Methods:**

In this feasibility study, a total of 44 participants were randomized to one of three conditions: full-spectrum CBD (*n* = 13, fsCBD - <0.3% THC), broad-spectrum CBD (*n* = 15, bsCBD – without THC), or placebo control (*n* = 16) for 8 weeks. The study was designed to assess the safety and tolerability of these treatments and to evaluate whether CBD demonstrated any clinical effects (Clinicaltrials.gov Identifier: NCT04873453; https://clinicaltrials.gov/study/NCT04873453). It was hypothesized that both CBD conditions would be well tolerated and would reduce drinking, alcohol dependence, and craving compared to placebo.

**Results:**

Analyses of attrition and side effect data indicated no significant differences across conditions, suggesting that both bsCBD and fsCBD were well tolerated. Individuals receiving fsCBD demonstrated reductions in craving but no reduction in drinks per drinking day.

**Discussion:**

In this pilot study, safety profiles fsCBD and bsCBD were similar, and fsCBD was associated with a greater reduction in craving and AUD symptoms relative to both bsCBD and placebo. Future studies with larger sample sizes will be necessary to replicate and extend these findings by addressing the question of whether a small amount of THC may work synergistically with CBD.

## 1 Introduction

Alcohol Use Disorder (AUD) is associated with socioeconomic costs approximating $250 billion in the United States alone and significantly contributes to increased mortality among working-age adults (1). Despite decades of research, currently available treatments for AUD are only moderately effective. Common interventions include psychosocial treatments (e.g., Cognitive Behavioral Therapy, Motivational Interviewing, Alcoholics Anonymous) and pharmacological treatments such as disulfiram, naltrexone, and acamprosate (2). Recent meta-analyses suggest that psychosocial treatments, particularly when combined with pharmacotherapy, are associated with improved substance use outcomes, with 12-month abstinence rates exceeding 40% in some cases (3, 4). However, treatment efficacy remains inconsistent, with significant variability in success rates across both psychosocial and pharmacological interventions (2, 5). Engagement and retention challenges (6), often driven by accessibility constraints (7), impact the effectiveness of behavioral therapies and have exhibited limited efficacy for severe cases (8, 9). Likewise, pharmacological treatments exhibit varying success rates, largely due to adverse effects (2, 10) and adherence issues (11–13). Given the modest efficacy of current therapies and treatments, there is an urgent need for novel treatment strategies that improve efficacy and address craving, relapse risk, and other public health concerns posed by AUD.

Cannabinoids like cannabidiol (CBD, a non-intoxicating substance) and delta-9-tetrahydrocannabinol (THC, an intoxicating substance) are naturally occurring active compounds found in cannabis that may offer alternative or adjunctive therapeutic options for AUD (14). Preclinical studies suggest that CBD modulates neurobiological pathways implicated in addiction, particularly those involving endocannabinoid and serotonergic systems regulating reward, stress, and impulsivity (15, 16). Evidence from animal studies suggests that CBD reduces drug- and alcohol-motivated behaviors (17). For example, CBD reduces the reinforcing properties of alcohol and decreases alcohol consumption frequency and drinking motivation (18, 19). CBD also attenuates cue- and stress-induced alcohol-seeking, reinstatement, anxiety, and impulsivity in animal models with and without a genetic proclivity to drink (17, 20, 21). CBD may also normalize withdrawal-associated behavior and gene expression induced by spontaneous alcohol withdrawal (22). However, the research evidence of CBD for AUD is somewhat mixed; for instance, a recent primate study found doses within the therapeutic dose range (5-40 mg/kg) did not significantly reduce alcohol-seeking or consumption in baboons (23). Although empirical human research is limited, recent studies found that CBD attenuates cue-elicited craving for opioids (24, 25), suggesting it may have broad benefits with respect to cue-elicited craving and addiction. A more recent investigation with alcohol found that 30 mg and 200 mg of CBD had minimal influence on breath alcohol content and a negligible influence on the stimulative and sedative effects of alcohol (26). These findings highlight the need for future research to elucidate the mechanisms and potential clinical utility of cannabinoids in AUD treatment.

Despite growing interest in cannabinoid-based interventions, few studies have examined the effect of commercially available products containing CBD, which are widely available without a prescription online and in retail outlets across North America. Given the widespread accessibility of CBD products, the public health significance of such research is high. CBD products are commonly classified as full-spectrum CBD comprising THC in quantities within the legal limits that define hemp (≤0.3%) and broad-spectrum CBD, which has no THC. In both formulations, minor cannabinoids and terpenes are present at relatively low concentrations, often <1 mg/g (27) or <1 mg/mL (28) depending on the distillation methods used. The objective of the present study was to examine the feasibility and effects of full-spectrum CBD (fsCBD, contains less than 0.3% THC) vs. broad-spectrum CBD (bsCBD, which does not contain THC) vs. a matched placebo (i.e., hemp seed oil comprising no CBD or THC) on alcohol use and craving in a population of individuals with moderate AUD. Participants were assessed over an 8-week-long treatment period with a post-treatment follow-up at 16 weeks. We hypothesized that both the fsCBD and bsCBD conditions would be associated with reduced craving and AUD symptoms at the end of treatment compared to placebo.

## 2 Materials and methods

### 2.1 Study design

This is a single-site, double-blind, placebo-controlled, parallel-group study evaluating the effectiveness of CBD-containing products compared to placebo in improving symptoms of alcohol use disorder (Clinicaltrials.gov Identifier: NCT04873453). The study was approved by the Colorado Multiple Institutional Review Board (COMIRB) at the University of Colorado Anschutz Medical Campus. Participants provided written informed consent before any study procedures were conducted. The study recruited and enrolled participants and completed procedures from September 2021 to July 2023. The CBD and placebo products were obtained from the supplier listed in the approved FDA IND: 153535 (Ecofibre Ananda Hemp, Georgetown, KY, USA).

### 2.2 Inclusion and exclusion criteria

Study inclusion criteria were as follows: 1) ages 21 to 60, 2) met the Diagnostic and Statistical Manual of Mental Disorders 5^th^ edition (DSM-5) criteria for current AUD of at least moderate severity, 3) currently seeking treatment for AUD or expressing a desire to reduce their drinking, 4) self-reported consuming at least 21 (male sex) or 14 (female sex) standard alcoholic drinks per week, and 5) self-reported consuming at least 5 (male) or 4 (female) standard alcoholic drinks in one day during the past seven days at the time of screening. Exclusion criteria were as follows: 1) self-reported current diagnosis of a substance use disorder other than alcohol, 2) self-reported nicotine use more than 5 times per day, 3) self-reported illicit substance use in the past 30 days or a positive urine drug test, 4) self-reported use of cannabis six or more times per month, 5) current or past treatment for a DSM-5 Axis I disorder, 6) endorsed current suicidal ideation with intent or plan, 7) currently using antiepileptic medications or medications known to affect alcohol intake or significantly interact with the only rigorously tested and FDA-approved purified CBD product in the U.S. (Epidiolex), 8) self-reported history of severe alcohol withdrawal, 9) clinically significant medical problems (e.g., cardiovascular, renal, gastrointestinal) that would impair participation or limit medication ingestion, 10) current or past alcohol-related medical illness (e.g., pancreatitis, hepatocellular disease), 11) liver enzymes (ALT and AST) greater than two times the upper limit of the normal range at baseline screening, and 12) females who are pregnant, nursing, or who are not using a reliable form of birth control.

### 2.3 Procedures

Individuals were recruited through traditional advertising and digital media outlets that included a link to an online eligibility screening survey. Those who met the initial eligibility criteria, including DSM-5 criteria for current AUD of at least moderate severity, were scheduled for an in-person baseline screening visit. During this visit, a research team member collected informed consent, health history information, vital signs, and a detailed list of current medications. The Mini-International Neuropsychiatric Interview (MINI) was used to assess the presence of psychiatric illnesses and to confirm AUD presence of at least moderate severity. The MINI was not used as a diagnostic tool, but rather, responses were evaluated by the study physician alongside self-report data, medical histories, and current medications to determine eligibility. A blood draw was used for a basic blood work-up (i.e., complete blood count and comprehensive metabolic panel), and a urine pregnancy screening was administered to participants able to become pregnant. A breathalyzer and urinalysis were conducted to confirm no recent alcohol or illicit drug use, with the exception of cannabis. The study physician, a licensed independent provider, reviewed all health-related information and made the final eligibility determination for each participant. Individuals deemed eligible to participate in the trial were randomized to a treatment condition and dispensed medication.

The treatment part of the trial lasted eight weeks (week 1 – week 8), and there was a follow-up visit two months after the end-of-treatment visit (i.e., week 16). Randomized participants attended in-person visits during weeks 4 and 8, completed online surveys during weeks 1-3 and 5-7, and concluded their participation in the trial with a week 16 post-treatment visit over Zoom. Clinical data and self-report measures were collected and managed in REDCap and collected at the three in-person visits (baseline, week 4, week 8) and week 16. Blood samples assessing cannabinoid and liver enzyme levels, and pregnancy screening were only collected at the three in-person visits. Safety assessments concerning side effects, changes in health status, and changes to medication were collected in the weekly surveys, at the three in-person visits, and also at the week 16 post-treatment follow-up visit. Safety assessments were collected at the week 16 visit to ensure no late-onset adverse effects and to monitor long-term safety. All in-person visits took place at the University of Colorado Anschutz Medical Campus.

### 2.4 Interventions

Participants were randomly assigned to one of three treatment groups: broad-spectrum CBD (bsCBD, CBD without THC), full-spectrum CBD (fsCBD, CBD with <0.3% THC), or a placebo control (hemp seed oil with no CBD or THC) for eight weeks. The CBD formulations were prepared by infusing hemp seed oil with approximately 15 mg of active cannabinoids per capsule (fsCBD: 15.19 mg CBD with 0.41 mg THC; bsCBD: 14.93 mg CBD with 0 mg THC) and encapsulated in a soft gelatin shell. The placebo capsules contained hemp seed oil without active cannabinoids. Capsules were identical in appearance across the three conditions. Cannabichromene (CBC) and cannabigerol (CBG) were present in concentrations of less than 0.01 mg in fsCBD and bsCBD, respectively. Seven of the 28 terpenes tested were present in concentrations of less than 0.01 mg in fsCBD with no detectable levels of terpenes in bsCBD or placebo.

Participants were instructed to take ten 15 mg capsules daily, totaling approximately 150 mg of CBD for those in the bsCBD and fsCBD groups or 150 mg of hemp seed oil for those in the placebo group. While no strict dosing schedule was required, participants were encouraged to distribute their intake throughout the day, with a suggested regimen of three capsules in the morning, three in the afternoon, and four in the evening. When the trial was initiated, the daily dose of 150 mg was chosen based on prior research suggesting minimal side effects (29, 30). Additionally, this dose was within a safe range for most individuals, with any side effects being generally mild and infrequent (31).

Participants were randomly assigned to a treatment group using the randomization module in the Research Electronic Data Capture (REDCap) system, a secure, web-based platform hosted at the University of Colorado (32). A statistician, independent of data collection, created a pre-generated randomization list before study initiation, which was uploaded into REDCap with assignments concealed from study personnel to maintain blinding. Study staff accessed the REDCap system to retrieve the next sequential randomization assignment (conditions labeled as A, B, or C), which was automatically generated based on the pre-generated randomization list in the randomization module. The system ensured real-time allocation assignment, preventing selection bias. Only the principal investigator had access to the full randomization sequence, while the study staff remained blinded throughout data collection and analysis.

### 2.5 Assessments

#### 2.5.1 Safety, tolerability and feasibility

##### 2.5.1.1 Adverse events and side effects

To evaluate the safety and tolerability of CBD, participants completed a weekly self-report survey throughout the study to document any health changes they experienced over the past week, such as stomach discomfort, mood fluctuations, or trouble sleeping. The survey included a general inquiry (e.g., “Have you noticed any health changes this week?”), a checklist of common issues (e.g., headache), and an open-response field for participants to provide additional details. Participants were instructed to report any changes, regardless of severity, treatment relevance, or positive/negative perception. Any adverse events occurring post-randomization to the study medication were coded and categorized using the Medical Dictionary for Regulatory Activities (MedDRA) System Organ Classes (SOC). Suicide risk was monitored by administering the Columbia-Suicide Severity Rating Scale (C-SSRS) each week during the eight-week treatment period and again at week 16. Any reports of suicidal ideation were followed up by a study psychiatrist.

Additionally, participants completed the 4-item PROMIS Fatigue 4a and PROMIS Sleep Disturbance 4a measures, which evaluated the intensity of fatigue and the quality of sleep over the previous seven days. Higher scores on these assessments corresponded to greater fatigue and poorer sleep quality. The summed raw scores were converted to T-scores, referenced to the U.S. general population, and rescaled with a mean of 50 and a standard deviation of 10.

##### 2.5.1.2 Liver enzymes

Liver enzymes alanine transaminase (ALT) and aspartate aminotransferase (AST) were monitored throughout the study using a comprehensive metabolic panel, which was conducted at baseline, week 4, and week 8.

##### 2.5.1.3 Compliance and blind integrity

To evaluate the feasibility of maintaining the blind, participants were asked at weeks 4 and 8 to guess which treatment condition they believed they had been assigned to. Condition identification accuracy was calculated as the percentage of correct responses, determined by dividing the total number of correct condition identifications by the total number of responses within that condition.

Medication adherence was assessed at weeks 4 and 8. To calculate compliance, the total number of capsules consumed was determined by counting the number of empty bottles (each containing 70 capsules) plus any remaining capsules in the partially used bottles. This total was then divided by the expected number of capsules consumed if the participant had been fully compliant since the previous visit (i.e., 10 capsules per day multiplied by the number of days between visits). Compliance percentages were calculated for each participant and then averaged within each condition to obtain the final compliance rate.

##### 2.5.1.4 Blood cannabinoid analysis

Blood levels of CBD and THC metabolites, 7-carboxy-cannabidiol (CBD-COOH) and delta-9 carboxy tetrahydrocannabinol (THC-COOH), were analyzed to verify participant compliance with study instructions. Certified phlebotomists collected venous blood at baseline and weeks 4 and 8. Plasma was stored at -80°C until processing using HPLC-MS/MS with an API5500 mass spectrometer. The assay’s lower limit of quantification is 500 pg/ml, with inter-assay precision within 85-115% and total imprecision below 15%, except at a lower limit.

#### 2.5.2 Primary alcohol outcomes

##### 2.5.2.1 Timeline follow-back: drinks per drinking day

The TLFB is a retrospective self-report method for tracking an individual’s substance use over a defined period (33). Participants were asked to recall the quantity and frequency of their alcohol consumption over the past 30 days. The average number of drinks consumed per drinking day (DPDD) in the last 30 days was calculated at baseline and weeks 4, 8, and 16.

##### 2.5.2.2 Alcohol dependence scale

The ADS is a 25-item questionnaire (α = .72) assessing alcohol use, including drinking patterns, cravings, tolerance, withdrawal, and health consequences (34). It was collected at baseline and weeks 4, 8, and 16. Items are scored from 0 (no problem) to 3 or 4 (significant problem), with a total score ranging from 0 to 47, where higher scores indicate greater dependence.

##### 2.5.2.3 Penn alcohol craving scale

The PACS is a 5-item measure (α = .84) assessing alcohol craving within the past week (35) and was collected at baseline and weeks 4, 8, and 16. Items are scored from 0 (no craving) to 6 (highest craving), with total scores ranging from 0 to 30, and higher scores indicate greater craving. PACS has been shown to better predict future drinking than the obsessive-compulsive drinking scale (OCDS) and the alcohol urge questionnaire (AUQ) (36).

#### 2.5.3 Secondary alcohol outcomes

##### 2.5.3.1 Alcohol use disorders identification test

The AUDIT is a 10-item questionnaire (α = .73) that screens for harmful alcohol use by assessing drinking behavior, psychological responses, and alcohol-related problems (37). Each item is from 0 to 4, with total scores ranging from 0 to 40, where higher scores indicate more hazardous drinking. The AUDIT was collected at baseline and weeks 4, 8, and 16.

##### 2.5.3.2 Impaired control scale-failed control

The ICS-FC is a 10-item measure (α = .79) assessing impulsivity, awareness of compulsion to drink, and control over drinking behavior (38). Items are scored from 0 to 4, with total scores ranging from 0 to 40, and where higher scores indicate more difficulty in controlling alcohol use (i.e., more failed attempts to control drinking). The ICS-FC was administered at baseline and weeks 4, 8, and 16.

### 2.6 Statistical analysis

The target sample size of 45 was selected to permit analysis of the primary research questions and to detect changes in AUD symptoms using G*Power statistical software with parameters at the two-tailed α of.05 and power level of.80. Group-level differences in baseline demographics and baseline scores on alcohol-related variables were assessed using analyses of variance (ANOVAs) and chi-squared tests. The prevalence and proportion of participants within each treatment group experiencing adverse events were compared using Fisher exact tests. To examine group-level differences in side effects (PROMIS fatigue and sleep disturbance), liver enzymes, blood cannabinoid levels and primary and secondary alcohol-related outcomes, intent-to-treat analyses were performed using linear mixed effects with maximum likelihood estimation of missing data and included all participants randomized to treatment (*N* = 44). Models concerning primary and secondary alcohol outcomes proceeded with 3 (4, 8, 16 weeks) by 3 (placebo, bsCBD, fsCBD) covarying for baseline scores. Models concerning liver enzymes and PROMIS measures of fatigue and sleep disturbance proceeded with 2 (4 and 8 weeks) by 3 (placebo, bsCBD, fsCBD) covarying for baseline scores. Lastly, the models concerning blood cannabinoid levels (CBD-COOH and THC-COOH) proceeded with 2 (4 and 8 weeks) by 3 (placebo, bsCBD, fsCBD) models, not covarying for baseline levels. Linear effects models were conducted using the software package Jamovi (version 2.4.14.0). Figures were generated in Prism Graph Pad (version 10.1.1). Results from the linear mixed effects analyses are presented below.

## 3 Results

### 3.1 Sample characteristics

Forty-four individuals were enrolled and randomized into three groups in a 1:1:1 ratio: bsCBD (*n* = 15), fsCBD (*n* = 13), and a matched placebo control (*n* = 16). No significant group-level differences in demographics and sample characteristics were observed (see **Table 1**). The majority of participants in each condition were Female (>90%), White (>90%), and non-Hispanic or Latino (>85%). The sample was primarily non-cannabis users, with only five individuals reporting past-month use at baseline: one in the placebo group (2 days) and two in each CBD condition (fsCBD and bsCBD), averaging 2.5 days in each group. See **Table 1** for additional information on sample demographics.

**Table 1:** Baseline demographics and sample characteristics.

Characteristic [% or Mean (Standard Deviation)]	Full-Spectrum CBD (fsCBD, *n*=13)	Broad-Spectrum CBD (bsCBD, *n*=15)	Hemp Seed Oil (Placebo, *n*=16)
Age	32.15 (1.98)	36.73 (1.85)	38.69 (1.79)
Sex (% Female)	92.31%	53.33%	81.25%
Race (% White)	92.31%	93.33%	93.75%
Ethnicity (% Non-Hispanic or Latino)	100.00%	93.33%	87.50%
Body Mass Index (BMI)	26.09 (2.01)	27.31 (1.94)	28.85 (1.88)
Baseline DPDD average	3.99 (.45)	4.59 (.42)	4.12 (.41)
Baseline ADS scores	12.69 (6.06)	13.07 (4.16)	11.44 (4.56)
Baseline PACS scores	12.69 (1.35)	11.8 (1.26)	14.06 (1.22)
Baseline AUDIT scores	17.08 (1.51)	18.47 (1.41)	17.06 (1.36)
Baseline ICS-FC scores	21.46 (1.52)	21.8 (1.42)	21.69 (1.37)
PROMIS Fatigue 4a			
Baseline	52.1 (1.45)	52.5 (1.11)	54.6 (1.3)
Week 4	54.3 (1.93)	52.5 (1.85)	49.1 (1.73)
Week 8	52 (1.93)	48.2 (1.9)	48.2 (1.78)
PROMIS Sleep Disturbance 4a			
Baseline	48 (1.68)	54.7 (1.17)	52.2 (.92)
Week 4	49.1 (1.81)	51.7 (1.74)	48.1 (1.58)
Week 8	51 (1.81)	48.7 (1.79)	48.3 (1.63)
Alanine transaminase (ALT) U/L^a^			
Baseline	17.5 (2.01)	16.7 (1.79)	28.9 (3.84)
Week 4	22.8 (3.22)	24.5 (3.01)	19.3 (3.04)
Week 8	22.2 (3.56)	25.4 (3.28)	21.2 (3.09)
Aspartate aminotransferase (AST) U/L^a^			
Baseline	20.4 (1.3)	19.3 (1.15)	25.2 (3.06)
Week 4	24 (2.19)	21.3 (2.03)	19.9 (1.99)
Week 8	23.2 (2.43)	23 (2.21)	21.9 (2.1)
Condition Identification Accuracy (%)^b^			
Week 4	66.67%	38.46%	73.33%
Week 8	66.67%	41.67%	57.14%
Compliance (%)^c^			
Week 4	97.50%	100.00%	99.30%
Week 8	90.00%	97.50%	95.40%

a Reference range: ALT (7-52 U/L) and AST (12-39 U/L).

b Percentages were calculated by dividing the number of correct responses by the total number of responses within each condition at weeks 4 and 8.

c Compliance (%) was determined by dividing the total number of capsules consumed by the expected number if fully compliant, then averaging per condition at weeks 4 and 8.

**Figure 1:**
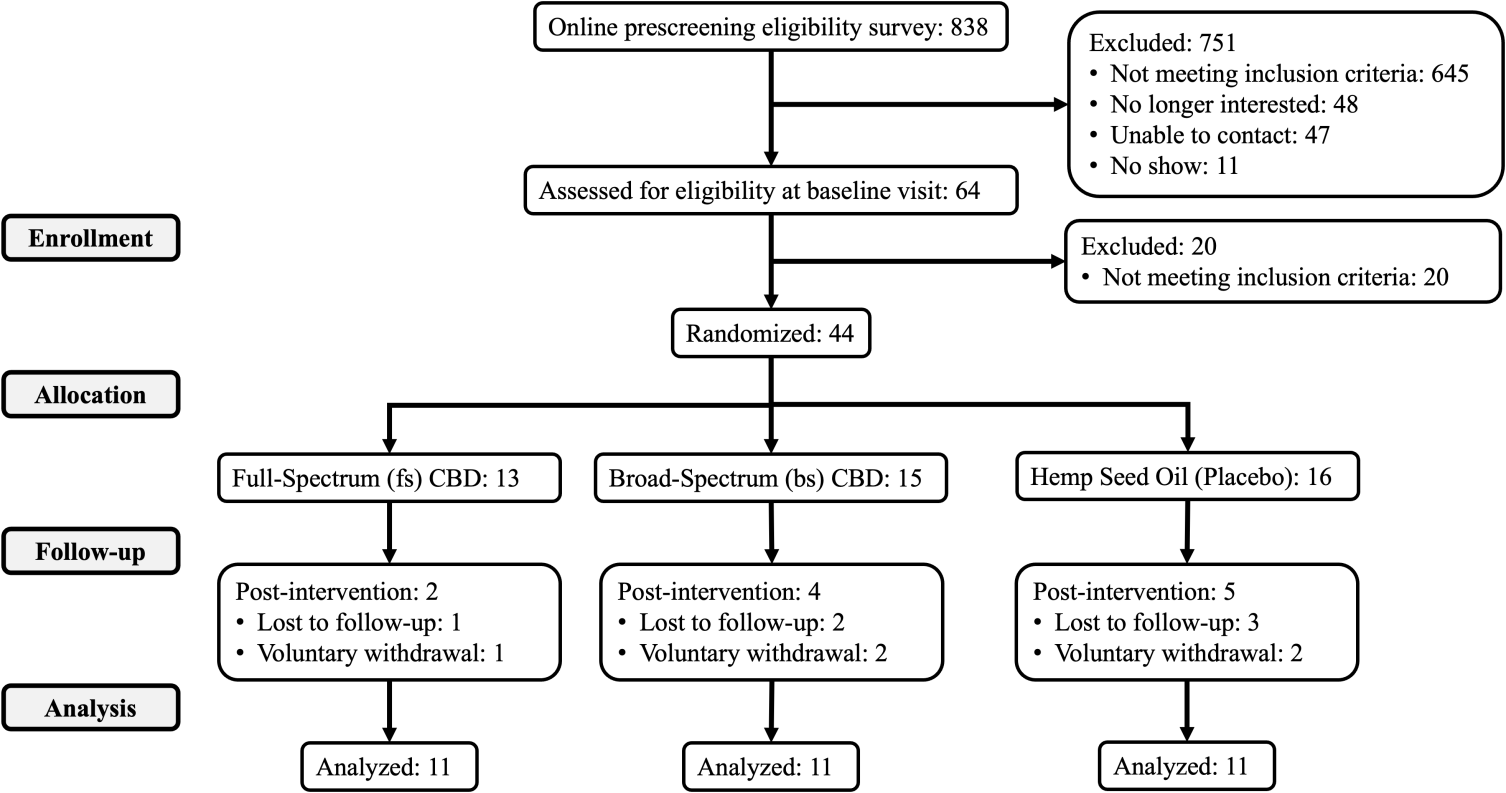
CONSORT diagram.

Of those randomized to treatment, 33 completed all study visits through week 16: bsCBD (*n* = 11), fsCBD (*n* = 11), and placebo (*n* = 11). Reasons for discontinuation were voluntary withdrawal from the study and lost to follow-up. Refer to CONSORT **Figure 1** for details. Five participants voluntarily withdrew (two in week 2, two in week 3, and one after week 4), all citing personal reasons, scheduling conflicts, or time constraints, with none withdrawing due to treatment-related side effects or adverse events.

### 3.2 Evaluation of intervention safety, tolerability, and feasibility

#### 3.2.1 Adverse events and suicidal ideation

The frequency and percentage of participants experiencing adverse events occurring during the 8-week study period are listed by MedDRA System Class (SOC) in **Supplementary Table 1**. A total of 190 adverse events were reported during the 8-week trial period: 71 in the fsCBD condition, 75 in the bsCBD condition, and 44 in the placebo condition. The most common adverse event was dry mouth, insomnia, and interrupted sleep in the fsCBD, bsCBD, and placebo conditions, respectively.

All adverse events (AEs) were assessed by the study physician as mild in severity, except for three unrelated to the intervention, which were classified as moderate. These included cases of COVID-19 symptoms, migraine, and musculoskeletal pain in the lumbar vertebrae. The average duration of sleep-related AEs (e.g., insomnia, early morning awakening, and interrupted sleep) in the bsCBD condition was 24 days, or approximately three weeks, during the intervention. Sleep-related AEs were slightly less common in the fsCBD group compared to bsCBD; however, when present, they lasted approximately five weeks, a duration similar to that observed in the placebo condition. Dry mouth in the fsCBD group persisted for most of the trial (approximately eight weeks). By the week 16 follow-up, 70% of reported AEs had resolved. Additionally, 90% of sleep-related and fatigue-related AEs were deemed possibly or probably related to the intervention.

No serious adverse events were reported in any group, and no participants endorsed thoughts of suicide on the C-SSRS during the trial, suggesting that neither fsCBD nor bsCBD was associated with suicidal ideation.

#### 3.2.2 PROMIS fatigue and sleep disturbance

Although CBD is commonly used to improve sleep, some data suggest negative effects, prompting us to use the PROMIS Fatigue and Sleep Disturbance questionnaires to assess whether CBD worsened sleep quality or increased fatigue intensity (i.e., daytime sleepiness). We found no significant differences in PROMIS Fatigue and Sleep Disturbance between conditions or over the course of the trial, suggesting that consuming 150 mg CBD daily did not negatively impact daytime sleepiness or sleep quality. See **Table 1** for descriptives.

#### 3.2.3 Liver enzymes (ALT and AST)

Lastly, there were no significant differences in the liver function enzymes ALT and AST between conditions or over the course of the trial, indicating that consuming 150 mg CBD daily did not negatively impact liver function. See **Table 1** for descriptives.

#### 3.2.4 Compliance and blind validity

To assess the validity of the blind, participants were queried on which treatment condition they believed they were randomly assigned at study weeks 4 and 8. Chi-squared tests indicated no relationship between condition assignment and guesses made (correct vs. incorrect) at weeks 4 (*χ* ^2^ = 3.846, *p* = .146) and 8 (*χ* ^2^ = 1.548, *p* = .461). See **Table 1** for descriptives.

#### 3.2.5 Blood cannabinoids (CBD-COOH and THC-COOH)

**Figure 2:**
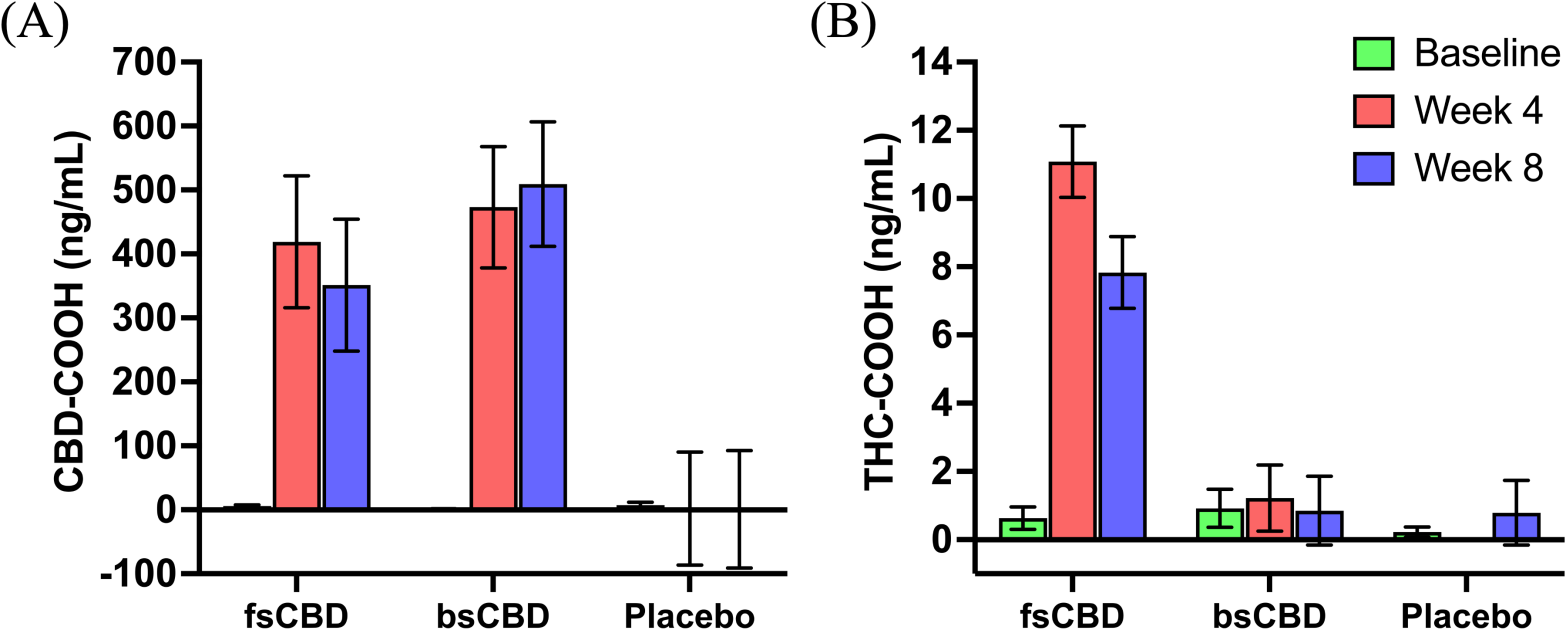
Blood cannabinoids. Mean blood levels (ng/mL) of **(A)** CBD-COOH and **(B)** THC-COOH by treatment condition (x-axis) at baseline (green), week 4 (orange), and week 8 (purple).

To assess adherence and confirm the effect of condition, blood metabolite levels (CBD-COOH and THC-COOH) were quantified at baseline and weeks 4 and 8. As expected, a significant main effect of condition on CBD-COOH (*F*_2,36_ = 8.592, *p* <.001) and THC-COOH levels (*F*_2,36_ = 29.11, *p* <.001) were observed. CBD-COOH was significantly higher in the CBD conditions relative to placebo at week 4 (bsCBD, *p* <.001; fsCBD, *p* = .004) and week 8 (bsCBD, *p* <.001; fsCBD, *p* = .015) and THC-COOH levels were significantly higher in the fsCBD condition compared to bsCBD and placebo at week 4 (bsCBD, *p* <.001; placebo, *p* <.001) and week 8 (bsCBD, *p* <.001; placebo, *p* <.001). No significant differences in CBD-COOH blood levels were found between the CBD conditions. See **Figure 2**.

### 3.3 Effects of CBD on alcohol-related outcomes

#### 3.3.1 Drinks per drinking day (TLFB)

The average number of drinks per drinking day in the past 30-days generated from the TLFB was calculated at baseline and weeks 4, 8, and 16. We did not observe a significant main effect of condition or time-by-condition interaction on drinks per drinking day. See **Table 1** for descriptives.

#### 3.3.2 Alcohol dependence (ADS)

There was no significant main effect of condition or time-by-condition interaction on the Alcohol Dependence Scale (ADS). There was a simple effect of condition where ADS scores significantly decreased in the bsCBD condition at week 16 compared to week 4 (*p* = .048) and week 8 (*p* = 0.26). ADS scores were also significantly lower in the bsCBD condition relative to placebo at week 8 (mean difference (*MD*) = 2.98, standard error (*SE*) = 1.34, *p* = .034). See **Table 1** for descriptives.

#### 3.3.3 Alcohol craving (PACS)

**Figure 3:**
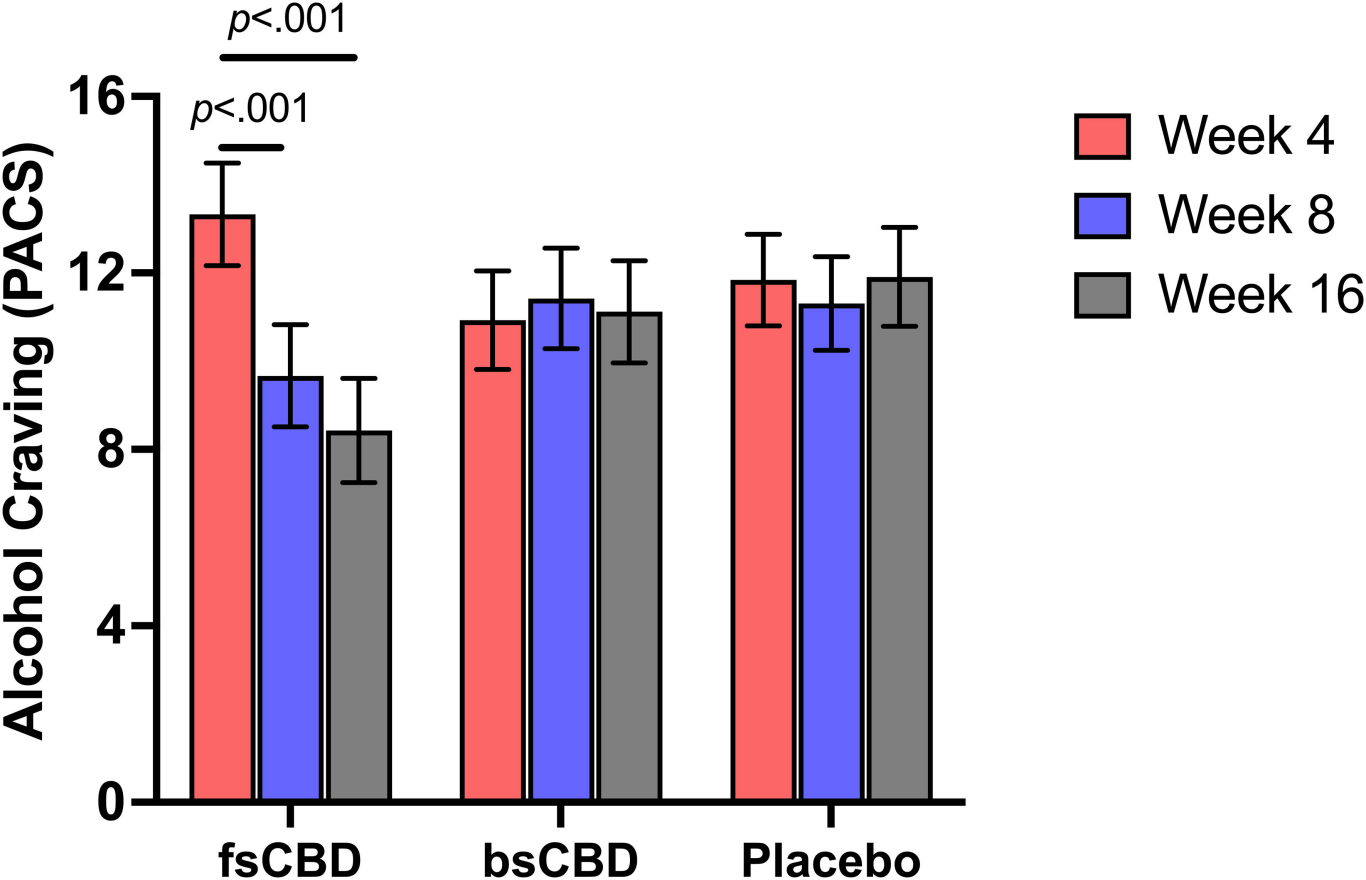
PACS scores (y-axis) at weeks 4 (orange), 8 (purple), and 16 (gray) by treatment condition (x-axis). Those assigned to fsCBD for eight weeks exhibited a greater decline in alcohol craving at 8 (*p* <.001) and 16 weeks (*p* <.001), while those assigned to bsCBD or placebo exhibited no change.

We observed a significant linear time-by-condition interaction on the Penn Alcohol Craving Scale (PACS) (*F*_4,35_ = 5.47, *p* <.001) where those in the fsCBD condition exhibited reduced alcohol craving at week 8 (*p* = .014) and week 16 (*p* <. 001) compared to placebo. Craving scores were also significantly lower in the fsCBD condition compared to placebo at week 16 (*MD* = 3.49, *SE* = 1.63, *p* = .037). No decreases were observed in the other two groups. See **Figure 3**.

#### 3.3.4 Harmful drinking (AUDIT)

**Figure 4:**
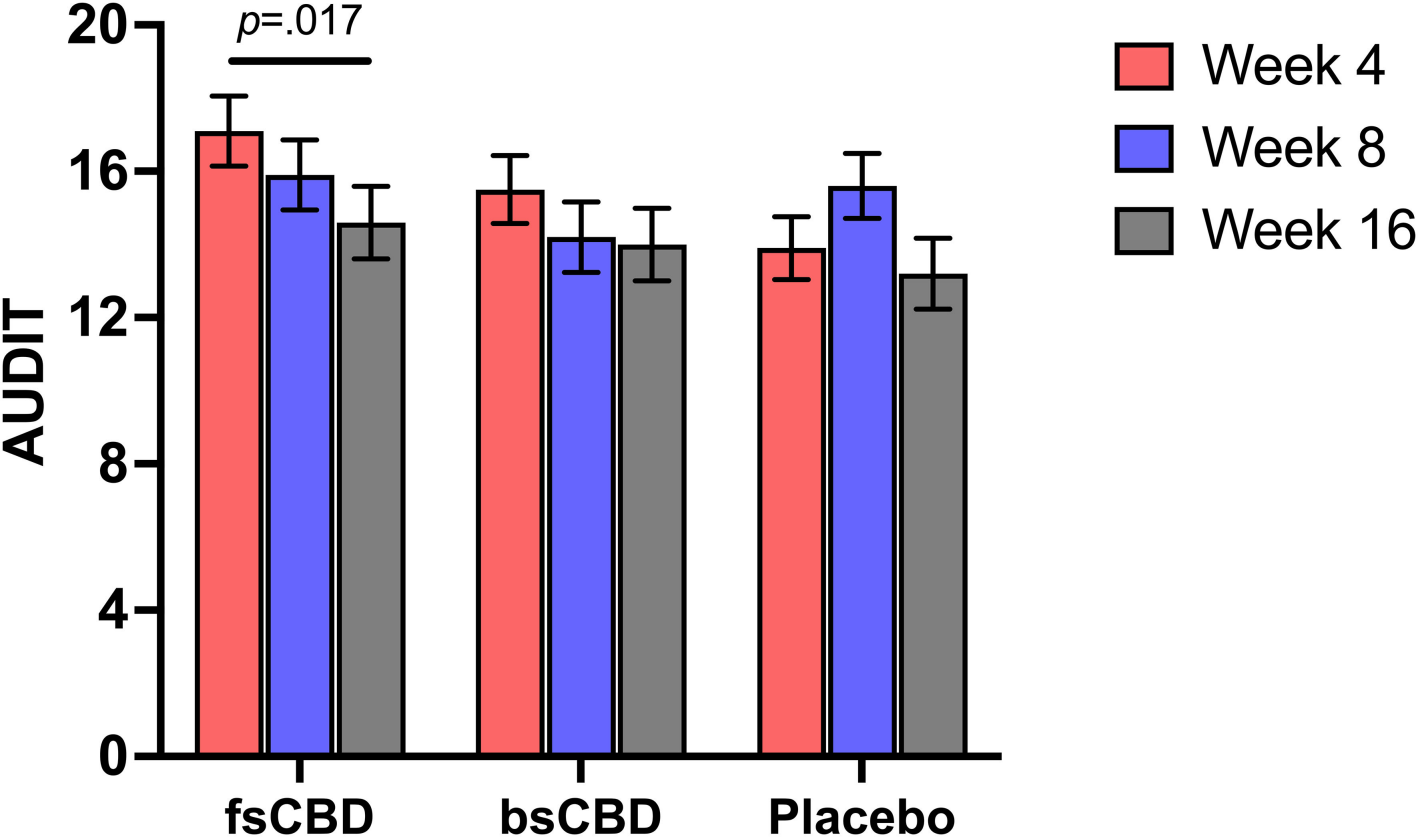
AUDIT scores (y-axis) at weeks 4 (orange), 8 (purple), and 16 (gray) by treatment condition (x-axis). Those assigned to fsCBD demonstrated a significant decrease in AUDIT scores from weeks 4 to 16 (*p* = .017).

There was not a significant time-by-condition interaction on the Alcohol Use Identification Test (AUDIT) (*F*_4,35_ = 1.751, *p* = .149). However, it was observed that AUDIT scores were significantly decreased at week 16 in the fsCBD condition relative to placebo (*MD* = 2.53, *SE* = 1.03, p = .017). See **Figure 4**.

#### 3.3.5 Control over drinking (ICS-FC)

**Figure 5:**
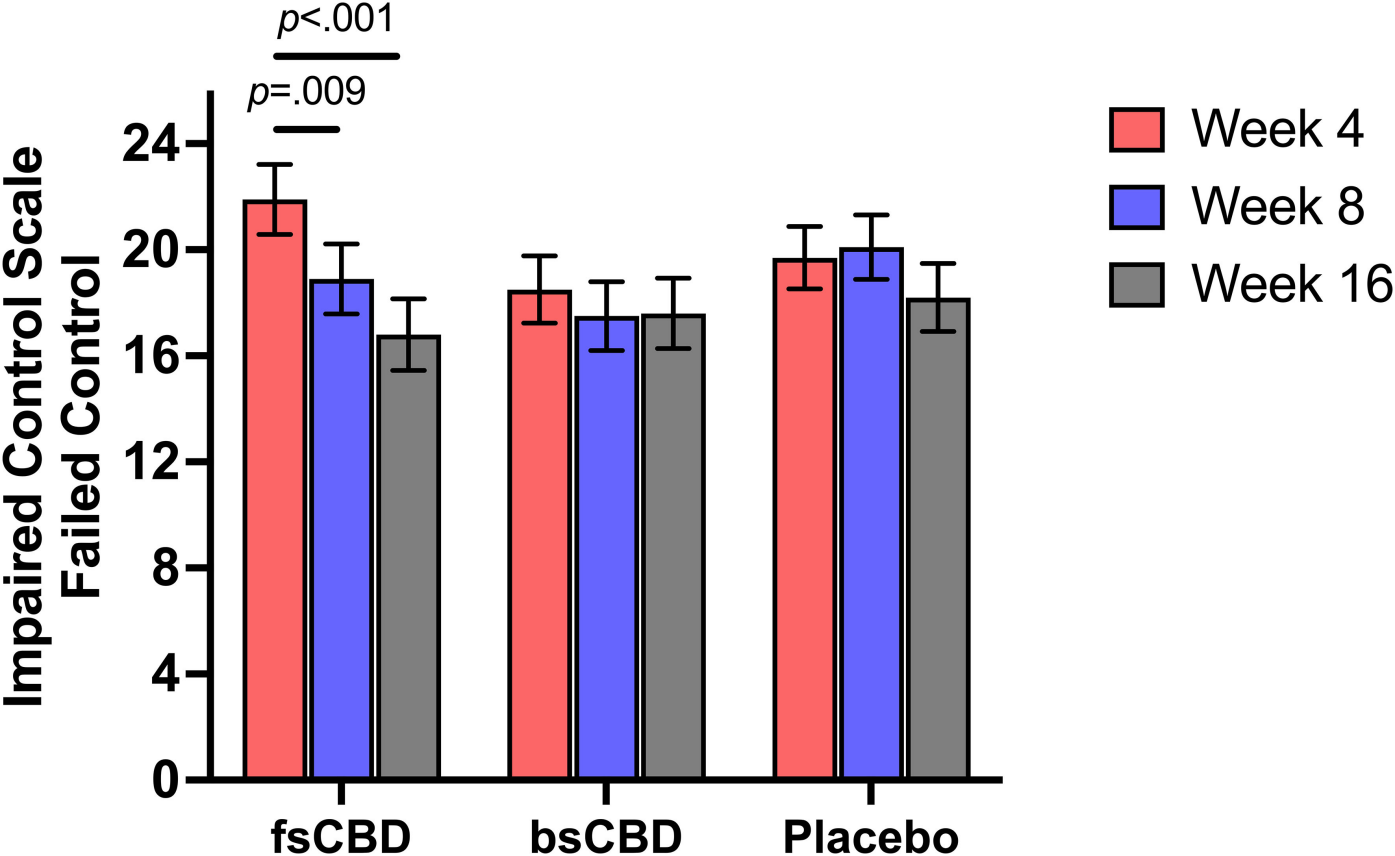
ICS-FC scores (y-axis) at weeks 4 (orange), 8 (purple), and 16 (gray) by treatment condition (x-axis). Individuals in the fsCBD exhibited a greater reduction in failed attempts to control drinking from weeks 4 to 8 (*p* = .009) and 4 to 16 (*p* <.001).

There was a significant linear time-by-condition interaction on the Impaired Control Scale-Failed Control (ICS-FC) (*F*_4,35_ = 2.539, *p* = .048), such that those in the fsCBD exhibited a greater decrease in failed attempts to control drinking at 8 weeks (*p* = .031) and 16 weeks (*p* = .028) relative to placebo. Further, simple effects revealed that within the fsCBD condition, failed attempts significantly declined from weeks 4 to 8 (*MD* = 2.92, *SE* = 1.09, *p* = .009) and weeks 4 to 16 (*MD* = 5.056, *SE* = 1.12, *p* <.001). See **Figure 5**.

## 4 Discussion

The results of this preliminary study suggest that daily doses of both bsCBD (150 mg) and fsCBD (150 mg) were safe and well tolerated in adults with moderate to severe AUD. Side effects were minimal, with no differences in liver enzymes, daytime sleepiness, or sleep quality across conditions. In addition, it is worth noting that the participants were unable to distinguish between placebo, bsCBD, and fsCBD at statistically greater than chance levels. While the results did not support a direct effect on drinks per drinking day, the results did suggest fsCBD reduced weekly craving, which is consistent with previous studies suggesting that CBD may have a beneficial effect in AUD models in mice (19) and rats (20). Results are also consistent with clinical studies suggesting that CBD may decrease craving for other substances of abuse (24). This is a small proof-of-concept study, and more extensive research is needed to confirm and extend these results to adequately understand the effects of CBD with and without THC in an AUD population.

The results highlight two priorities for future research. Specifically, the finding that CBD combined with a small amount of THC demonstrated a stronger improvement in AUD symptoms relative to bsCBD without THC requires replication and studies that can identify the underlying mechanisms. A previous study had similarly observed that a small amount of THC combined with CBD was associated with less alcohol use compared to those using cannabis with high THC concentrations or cannabis with equivalent amounts of THC and CBD (39). The findings in the present study are consistent with the former study, suggesting that a small amount of THC may have a beneficial effect in reducing craving and other AUD symptoms. However, more research is needed to evaluate different doses and ratios of CBD and THC. Clearly, an essential step in this research is to evaluate a range of doses for both bsCBD and fsCBD. For fsCBD, the dose range will be limited by the overall amount of THC. Future studies should test a broader range of tolerable doses to understand better how CBD and THC interact.

Although the primary aim of this pilot study was to assess the practicality of our methods (i.e., feasibility, scalability) and intervention tolerability, the study has several limitations. First, the small sample size necessitates further validation in a larger, independent cohort. Additionally, our recruitment method differs from traditional clinical trials, where patients are enrolled following a medical consultation for AUD diagnosis and treatment, which may have contributed to the low eligibility rate. Another limitation is the relatively low CBD dose used in this study compared to other trials, which may have influenced the findings. Furthermore, while pure CBD formulations are often used to isolate the effects of CBD alone, this study aimed to evaluate commercially available CBD products with formulations containing trace amounts of minor cannabinoids and terpenes, making it difficult to isolate the effects of CBD alone. However, given their extremely low concentrations and limited bioavailability, their impact is likely minimal. Additionally, relying on the TLFB for substance use tracking, rather than a daily tracker or diary, may have introduced inaccuracies in self-reported drinking behavior. Lastly, we recognize treatment expectancies may have influenced self-reported recall of past and current drinking behavior.

In conclusion, further research on the effects of cannabinoids in AUD is clearly needed. The present results suggest good tolerability, low potential for adverse effects, and potential clinical efficacy, supporting the rationale for additional investigations. It is important to note that this area of research has important public health implications, not only because the field lacks new effective treatments but also because products with CBD are already widely utilized over-the-counter by consumers across the United States despite the limited formal evidence regarding their safety and efficacy. Future studies should prioritize large, well-powered RCTs to validate these preliminary findings in individuals with varying AUD severity and treatment histories. Comparative trials examining different CBD formulations and doses are important for determining optimal therapeutic levels, given this study administered relatively low doses. Future studies should also examine the impact of low-dose THC and other minor cannabinoids in fsCBD versus bsCBD formulations on alcohol use outcomes. By taking these considerations into account, future research could clarify the underlying mechanisms of these effects and advance our understanding of the potential utility of cannabinoids in the treatment of AUD.

## Data Availability

The raw data supporting the conclusions of this article will be made available by the authors, without undue reservation.
