# Supplementary material for: A preliminary randomized trial of the safety, tolerability, and clinical effects of hemp-derived cannabidiol in alcohol use disorder
Source: Front Psychiatry. 2025 Apr 28;16:1516351. doi: 10.3389/fpsyt.2025.1516351 (PMC12066606; doi:10.3389/fpsyt.2025.1516351)
Supplement: Supplementary file 1 [file Table1.docx]

**Supplemental Table 1.** Frequency and percentage of participants experiencing adverse events (AEs) after randomization to study medication through the end-of-treatment visit. AEs are organized by MedDRA System Class (SOC) and preferred term (PT). The proportion of participants experiencing AEs within each treatment condition was compared using Fisher exact tests.

| SOC |  |  | fsCBD (n=13) | fsCBD  (%) |  | bsCBD (n=15) | bsCBD ( %) |  | Placebo (n=16) | Placebo  (%) |  | Total (N=44) | Total (%) |  | p-value |
| --- | --- | --- | --- | --- | --- | --- | --- | --- | --- | --- | --- | --- | --- | --- | --- |
| Ear and labyrinth disorders | | | | | | | | | | | | | | | |
|  | Ear pain |  | 1 | 7.7 |  | 0 | 0.0 |  | 0 | 0.0 |  | 1 | 2.3 |  | 0.295 |
| Eye disorders | | | | | | | | | | | | | | | |
|  | Eye Irritation |  | 0 | 0.0 |  | 1 | 6.7 |  | 1 | 6.3 |  | 2 | 4.5 |  | 1.000 |
|  | Stye |  | 0 | 0.0 |  | 1 | 6.7 |  | 0 | 0.0 |  | 1 | 2.3 |  | 0.636 |
|  | Vision blurred |  | 0 | 0.0 |  | 1 | 6.7 |  | 0 | 0.0 |  | 1 | 2.3 |  | 0.636 |
| Gastrointestinal disorders | | | | | | | | | | | | | | | |
|  | Constipation |  | 1 | 7.7 |  | 1 | 6.7 |  | 2 | 12.5 |  | 4 | 9.1 |  | 1.000 |
|  | Diarrhea |  | 0 | 0.0 |  | 0 | 0.0 |  | 1 | 6.3 |  | 1 | 2.3 |  | 1.000 |
|  | Dry mouth |  | 6 | 46.2 |  | 0 | 0.0 |  | 2 | 12.5 |  | 8 | 18.2 |  | 0.005 |
|  | Dyspepsia |  | 0 | 0.0 |  | 1 | 6.7 |  | 0 | 0.0 |  | 1 | 2.3 |  | 0.636 |
|  | Flatulence |  | 0 | 0.0 |  | 1 | 6.7 |  | 1 | 6.3 |  | 2 | 4.5 |  | 1.000 |
|  | Hemorrhoids |  | 0 | 0.0 |  | 1 | 6.7 |  | 0 | 0.0 |  | 1 | 2.3 |  | 0.636 |
|  | Gastrointestinal pain |  | 2 | 15.4 |  | 0 | 0.0 |  | 1 | 6.3 |  | 3 | 6.8 |  | 0.281 |
|  | Nausea |  | 1 | 7.7 |  | 1 | 6.7 |  | 1 | 6.3 |  | 3 | 6.8 |  | 1.000 |
| General disorders and administration site conditions | | | | | | | | | | | | | | | |
|  | Chest pain |  | 0 | 0.0 |  | 1 | 6.7 |  | 0 | 0.0 |  | 1 | 2.3 |  | 0.636 |
|  | Fatigue |  | 6 | 46.2 |  | 5 | 33.3 |  | 3 | 18.8 |  | 14 | 31.8 |  | 0.296 |
|  | Flu like symptoms |  | 1 | 7.7 |  | 0 | 0.0 |  | 0 | 0.0 |  | 1 | 2.3 |  | 0.295 |
|  | Subjective high feeling after dosing |  | 0 | 0.0 |  | 1 | 6.7 |  | 0 | 0.0 |  | 1 | 2.3 |  | 0.636 |
| Infections and infestations | | | | | | | | | | | | | | | |
|  | COVID-19 |  | 1 | 7.7 |  | 0 | 0.0 |  | 3 | 18.8 |  | 4 | 9.1 |  | 0.245 |
|  | Gastrointestinal illness |  | 1 | 7.7 |  | 0 | 0.0 |  | 0 | 0.0 |  | 1 | 2.3 |  | 0.295 |
| Investigations | | | | | | | | | | | | | | | |
|  | Heart rate increased |  | 0 | 0.0 |  | 1 | 6.7 |  | 1 | 6.3 |  | 2 | 4.5 |  | 1.000 |
|  | Heart rate irregular |  | 1 | 7.7 |  | 0 | 0.0 |  | 0 | 0.0 |  | 1 | 2.3 |  | 0.295 |
|  | Weight loss |  | 1 | 7.7 |  | 0 | 0.0 |  | 1 | 6.3 |  | 2 | 4.5 |  | 0.746 |
|  | Weight gain |  | 2 | 15.4 |  | 0 | 0.0 |  | 1 | 6.3 |  | 3 | 6.8 |  | 0.281 |
| Metabolism and nutrition disorders | | | | | | | | | | | | | | | |
|  | Decreased appetite |  | 2 | 15.4 |  | 1 | 6.7 |  | 0 | 0.0 |  | 3 | 6.8 |  | 0.187 |
|  | Increased appetite |  | 3 | 23.1 |  | 2 | 13.3 |  | 0 | 0.0 |  | 5 | 11.4 |  | 0.139 |
|  | Increased thirst |  | 3 | 23.1 |  | 1 | 6.7 |  | 1 | 6.3 |  | 5 | 11.4 |  | 0.412 |
| Musculoskeletal and connective tissue disorders | | | | | | | | | | | | | | | |
|  | Musculoskeletal pain |  | 0 | 0.0 |  | 1 | 6.7 |  | 0 | 0.0 |  | 1 | 2.3 |  | 0.636 |
|  | Myalgia |  | 2 | 15.4 |  | 1 | 6.7 |  | 0 | 0.0 |  | 3 | 6.8 |  | 0.187 |
| Nervous system disorders | | | | | | | | | | | | | | | |
|  | Carpal tunnel syndrome |  | 0 | 0.0 |  | 0 | 0.0 |  | 1 | 6.3 |  | 1 | 2.3 |  | 1.000 |
|  | Concentration impairment |  | 3 | 23.1 |  | 0 | 0.0 |  | 0 | 0.0 |  | 3 | 6.8 |  | 0.022 |
|  | Dizziness |  | 1 | 7.7 |  | 1 | 6.7 |  | 0 | 0.0 |  | 2 | 4.5 |  | 0.526 |
|  | Dysgeusia |  | 0 | 0.0 |  | 1 | 6.7 |  | 0 | 0.0 |  | 1 | 2.3 |  | 0.636 |
|  | Headache |  | 2 | 15.4 |  | 5 | 33.3 |  | 3 | 18.8 |  | 10 | 22.7 |  | 0.547 |
|  | Memory impairment |  | 1 | 7.7 |  | 2 | 13.3 |  | 0 | 0.0 |  | 3 | 6.8 |  | 0.384 |
|  | Somnolence |  | 4 | 30.8 |  | 1 | 6.7 |  | 1 | 6.3 |  | 6 | 13.6 |  | 0.184 |
|  | Syncope |  | 1 | 7.7 |  | 1 | 6.7 |  | 0 | 0.0 |  | 2 | 4.5 |  | 0.526 |
| Psychiatric disorders | | | | | | | | | | | | | | | |
|  | Agitation |  | 1 | 7.7 |  | 7 | 46.7 |  | 1 | 6.3 |  | 9 | 20.5 |  | 0.011 |
|  | Anxiety |  | 2 | 15.4 |  | 6 | 40.0 |  | 1 | 6.3 |  | 9 | 20.5 |  | 0.075 |
|  | Confusion |  | 0 | 0.0 |  | 1 | 6.7 |  | 0 | 0.0 |  | 1 | 2.3 |  | 0.636 |
|  | Depression |  | 3 | 23.1 |  | 3 | 20.0 |  | 1 | 6.3 |  | 7 | 15.9 |  | 0.350 |
|  | Early Morning Awakening |  | 4 | 30.8 |  | 5 | 33.3 |  | 2 | 12.5 |  | 11 | 25.0 |  | 0.383 |
|  | Insomnia |  | 4 | 30.8 |  | 10 | 66.7 |  | 2 | 12.5 |  | 16 | 36.4 |  | 0.007 |
|  | Interrupted Sleep |  | 2 | 15.4 |  | 7 | 46.7 |  | 7 | 43.8 |  | 16 | 36.4 |  | 0.174 |
| Renal and urinary disorders | | | | | | | | | | | | | | | |
|  | Other, Kidney stones |  | 0 | 0.0 |  | 0 | 0.0 |  | 0 | 0.0 |  | 0 | 0.0 |  | 0.174 |
|  | Urinary retention |  | 1 | 7.7 |  | 0 | 0.0 |  | 0 | 0.0 |  | 1 | 2.3 |  | 0.295 |
| Reproductive system and breast disorders | | | | | | | | | | | | | | | |
|  | Dysmenorrhea |  | 1 | 7.7 |  | 0 | 0.0 |  | 0 | 0.0 |  | 1 | 2.3 |  | 0.295 |
|  | Testicular pain |  | 0 | 0.0 |  | 1 | 6.7 |  | 0 | 0.0 |  | 1 | 2.3 |  | 0.636 |
| Respiratory, thoracic and mediastinal disorders | | | | | | | | | | | | | | | |
|  | Cough |  | 0 | 0.0 |  | 1 | 6.7 |  | 2 | 12.5 |  | 3 | 6.8 |  | 0.764 |
|  | Laryngeal inflammation |  | 3 | 23.1 |  | 0 | 0.0 |  | 0 | 0.0 |  | 3 | 6.8 |  | 0.022 |
|  | Nasal congestion |  | 2 | 15.4 |  | 1 | 6.7 |  | 3 | 18.8 |  | 6 | 13.6 |  | 0.652 |
| Skin and subcutaneous tissue disorders | | | | | | | | | | | | | | | |
|  | Skin rash |  | 2 | 15.4 |  | 0 | 0.0 |  | 1 | 6.3 |  | 3 | 6.8 |  | 0.281 |
